# A case of racemose and intraventricular neurocysticercosis in an unusual location

**DOI:** 10.4102/sajr.v25i1.2171

**Published:** 2021-12-06

**Authors:** Priya Singh, Surya P. Singh

**Affiliations:** 1 Department of Radiodiagnosis, Sanjay Gandhi Postgraduate Institute of Medical Science, Lucknow, India; 2 Department of Radiodiagnosis, All India Institute of Medical Sciences (AIIMS), New Delhi, India

**Keywords:** neurocysticercosis, racemose neurocysticercosis, grape-like clusters, hydrocephalus, intraventricular neurocysticercosis

## Abstract

Racemose and intraventricular neurocysticercosis are uncommon types of neurocysticercosis, resulting in a multiloculated, grape-like cluster appearance in the cerebrospinal fluid (CSF) spaces. A male patient presented with symptoms of raised intracranial pressure and demonstrated racemose neurocysticercosis at an atypical location involving the region of the crus of the fornix at the level of the body of lateral ventricles on magnetic resonance imaging. Associated intraventricular neurocysticercosis was seen in the atrium of the left lateral ventricle and fourth ventricle.

## Introduction

Neurocysticercosis is the most common parasitic infection of the central nervous system. It is more prevalent in Latin America, Asia and Africa. It can affect the brain parenchyma, subarachnoid spaces and ventricles. In racemose neurocysticercosis, cysticerci are located in basal cisterns, subarachnoid spaces and ventricles producing multiloculated cystic lesions with mass effect and a surrounding inflammatory reaction.^1^ Previously reported cases of racemose neurocysticercosis demonstrated these lesions mostly in the basal cisterns, Sylvian fissure and a few of them in anterior interhemispheric fissure.^2,3,4,5^ This report describes an unusual location of racemose neurocysticercosis located anterior and inferior to the splenium of the corpus callosum and between the fornixes associated with intraventricular neurocysticercosis in the atrium of the left lateral ventricle and fourth ventricle.

## Case history

A 34-year male presented with a history of gradually progressive headache, nausea, vomiting and dizziness for 15 days. There was no history of seizures or visual symptoms and neurological examination did not reveal any focal neurological deficits or signs of meningitis.

Initially, a non-contrast CT scan was performed, which revealed moderate dilatation of both lateral ventricles, as well as the third and fourth ventricles with a few cystic lesions in the forniceal region. No calcified lesions were appreciated at CT imaging.

**FIGURE 1:**
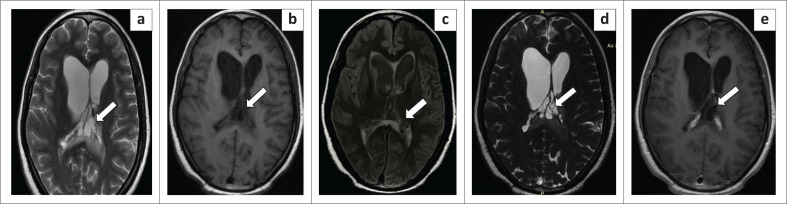
Magnetic resonance imaging of the brain in the axial plane. T2W(a), T1W (b), fluid attenuated inversion recovery (c), 3-D constructive interference in steady-state (d), T1 post-contrast (e) images reveal multiple small T2 hyperintense, T1 hypointense cystic lesions (white arrows) clustered together in the region of the crus of the fornix, anterior to the splenium of the corpus callosum. These lesions were suppressed on fluid attenuated inversion recovery and followed cerebrospinal fluid signal intensity. There was no enhancement of the cyst with no eccentric enhancing dot within the cyst. Axial 3-D constructive interference in steady-state magnetic resonance (d) image better delineates the cystic lesions producing a grape cluster-like appearance.

**FIGURE 2:**
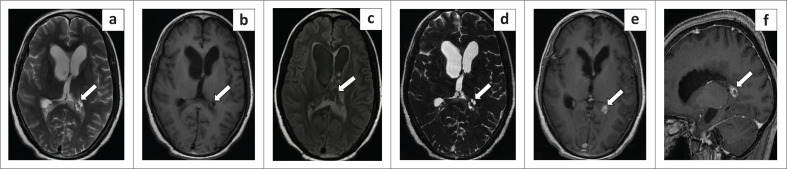
Magnetic resonance imaging of the brain. Axial T2 weighted (a), T1 weighted (b), fluid attenuated inversion recovery (c), 3-D constructive interference in steady-state (d), T1 post-contrast (e) and (f) sagittal magnetic resonance imaging 3-D T1 weighted post-contrast images demonstrate a T2 hyperintense, T1 hypointense lobulated multi-cystic lesion (white arrow) in the atrium of the left lateral ventricle. The lesion supresses on fluid attenuated inversion recovery and reveals post-contrast enhancement of the cyst wall with no eccentric enhancing foci. Cystic lesions were better delineated on the axial 3-D constructive interference in steady-state magnetic resonance image (d).

**FIGURE 3:**
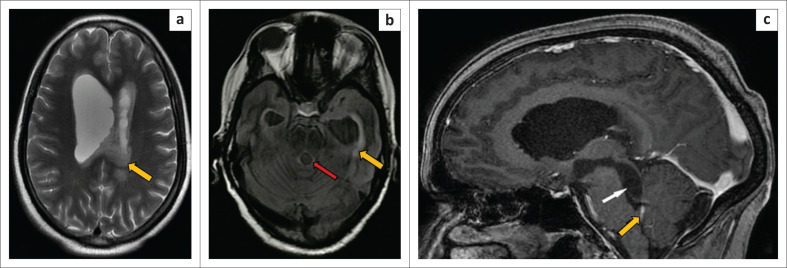
Magnetic resonance imaging of the brain. Axial T2 weighted (a) and fluid attenuated inversion recovery (b) images indicate asymmetrically dilated lateral ventricles (yellow arrows) and fourth ventricle (red arrow) with surrounding periventricular signal. Sagittal magnetic resonance imaging 3-D spoiled gradient recalled echo sequence (SGPGR BRAVO) post-contrast sequence image (c) demonstrates the dilated lateral ventricle, third ventricle, aqueduct of Sylvius and fourth ventricle. An abrupt narrowing was observed at the obex of the fourth ventricle (yellow arrow). A thin membrane-like structure (white arrow) was seen in the fourth ventricle, which possibly represented non-enhancing intraventricular cyst or a post-inflammatory membrane in the fourth ventricle, causing obstruction and upstream dilatation.

For further evaluation, MRI with contrast was acquired using a 3 tesla (T) magnetic resonance (MR) scanner at our institute. Magnetic resonance imaging revealed multiple small clustered thin-walled cystic lesions in the region of crus of fornix at the level of the body of lateral ventricles. The lesions were hypointense on T1 weighted imaging (WI), hyperintense on T2WI and revealed suppression on fluid attenuated inversion recovery (FLAIR) sequence images. No post-contrast enhancement of the cyst wall was seen. Surrounding parenchymal T2 and FLAIR hyperintensity was observed (Figure 1). Multiple similar small clustered cysts were also noticed in the atrium of the left lateral ventricle with enhancement of the cyst walls on post-contrast T1W images (Figure 2). Cyst walls were more conspicuous on the 3-D constructive interference in steady-state (CISS) sequence (Figures 1d and 2d). The ventricles were moderately dilated and associated with increased periventricular T2 and FLAIR signal intensity (Figures 3a and 3b). Ventricular dilatation extended up to the level of obex of the fourth ventricle. A few thin membranes or septations were seen in the fourth ventricle on the 3-D spoiled gradient recalled echo (SGPGR BRAVO) T1 post-contrast sequence, obstructing the outlet of the fourth ventricle (Figure 3c). However, these intraventricular membranes were not well delineated on other conventional sequences. No eccentric dot was seen within any of these lesions. No other focal brain parenchymal lesions were noticed. There were no blooming foci seen on susceptibility-weighted imaging (SWI).

Based on these MRI findings, a presumptive diagnosis of racemose and intraventricular neurocysticercosis with obstructive hydrocephalus was made. Differentials considered were ependymal cyst, choroid plexus cyst and colloid cyst. The possibility of an intraventricular neurocysticercosis cyst or post-inflammatory membrane was considered for the fourth ventricle lesion causing upstream obstruction. The patient underwent right ventriculoperitoneal shunting. Cerebrospinal fluid (CSF) was evaluated with enzyme-linked immunoassay (ELISA) was positive for neurocysticercosis. The patient was treated with albendazole and steroids and follow-up imaging was advised.

## Discussion

Neurocysticercosis is endemic in many developing countries, especially in Latin America, South-east Asia, the Indian subcontinent and sub-Saharan Africa. With increasing immigration and travel, the incidence of neurocysticercosis is also growing in developed countries. True disease prevalence is not well known because of the scarcity of case notification systems, systematic population studies and the absence of a gold standard test for screening and detection of asymptomatic disease. Data from different studies have shown that the prevalence of human taeniasis varies from 2.5% to 8.0% in India and the disease burden of neurocysticercosis causing active epilepsy is about one per 1000 population.^6^

Neurocysticercosis is a parasitic disease caused by Taenia solium. Humans are the only known definitive host for the organism and neurocysticercosis occurs when the human acts as an intermediate host after ingestion of its egg present in food contaminated with faeces of the tapeworm carrier. No inflammatory response is elicited when the cyst wall is intact, but when the parasite dies, then cyst contents are released, causing oedema and surrounding inflammatory changes.^7,8^ Neurocysticercosis is classified based on its location, namely subarachnoid-cisternal space, parenchymal, ventricles, spinal and mixed form.^8^ These different forms may also co-exist. Parenchymal is the most common location where it can be seen in any of the four stages, namely vesicular, colloid vesicular, granular nodular and nodular calcified.^8^

Cysticerci can reach the ventricles through the choroid plexus, moving freely within the ventricles or attaching to the ependyma. From the ventricles, it can travel to the basal cisterns, cerebral and spinal subarachnoid spaces and become lodged in these regions. The most common site of intraventricular neurocysticercosis is the fourth ventricle, followed by the third ventricle, lateral ventricle and cerebral aqueduct, respectively.^9^ Racemose neurocysticercosis is a type of extra-parenchymal neurocysticercosis characterised by the rapid growth of cyst walls forming grape-like clusters associated with degenerating scolices.

Bickerstaff et al. suggested that in cisterns, there is no limiting host response of encapsulation leading to uncontrolled proliferation.^10^ In different studies, the presence of the racemose variant is reported in 15.0% – 54.0% of patients.^1^ A study by Sierra et al., evaluated 125 extra parenchymal neurocysticercosis cases in which 51.6% were in basal subarachnoid cisterns, 33.7% in the ventricular system, 12.5% in Sylvian fissure and 2.2% in subarachnoid space of the medulla.^11^ Spinal neurocysticercosis is the rarest type of neurocysticercosis, which is most commonly associated with concomitant intracranial neurocysticercosis. Clinical symptoms depend on the stage of the lesions, their location and the host immune response.

Neurocysticercosis is one of the most common causes of recurrent seizures in both the parenchymal and subarachnoid forms because of oedema and encephalitis.^12^ In the extra parenchymal form, degeneration elicits an inflammatory response, causing arachnoiditis and ventriculitis.^13^ Hydrocephalus may also develop because of mass effect of the cyst, arachnoiditis (in subarachnoid neurocysticercosis) and ventriculitis (intraventricular neurocysticercosis), leading to adhesions and obstruction.^13^ An intraventricular cyst may be freely mobile, or it can attach to the ependymal wall of the ventricle. If a cyst is freely mobile in the ventricular cavities, it can cause Bruns syndrome characterised by episodes of sudden headaches, papilloedema and loss of consciousness, developing upon cephalic movement and followed by rapid clinical recovery.^14^ It develops as a result of intermittent obstructive hydrocephalus caused by a freely mobile intraventricular cyst that acts as a ball-valve mechanism.^14^ Ependymitis following cyst degeneration may also lead to intraventricular loculation, which is often challenging to treat. Inflammatory response surrounding subarachnoid neurocysticercosis may progress to vasculitis resulting in cerebrovascular accidents.^15^ Spinal neurocysticercosis can present with paresis, paralysis and polyradiculopathy.

The diagnosis is confirmed by neuroimaging and serological tests. Computed tomography (CT) is the initial imaging study that can identify active neurocysticercosis and the calcified stage of the disease. However, identifying racemose and intraventricular neurocysticercosis is difficult on CT because the cysts are iso-dense to CSF and usually non-enhancing. Magnetic resonance imaging with contrast is the imaging modality of choice for neurocysticercosis.^8^ Magnetic resonance imaging can show the pathological evolution of the disease, namely the vesicular, colloidal, granular nodular and calcified nodular stages. Typical neurocysticercosis lesions are smooth ring enhancing cystic lesions with an eccentric enhancing dot representing the scolex.

Racemose neurocysticercosis is mainly seen around the rostral brainstem or Sylvian fissure and usually lacks a scolex with mild or no surrounding oedema.^1^ Asymmetrically widened subarachnoid spaces and ventricles in a known case of cysticerci or neurocysticercosis can be a clue to the presence of racemose neurocysticercosis. T2 weighted imaging and FLAIR sequences in the racemose variant show multiloculated clustered cystic lesions in the subarachnoid spaces that follow CSF signal intensity and exert mass effect on surrounding structures.^8^ Surrounding wall enhancement may occasionally be seen in racemose neurocysticercosis because of associated arachnoiditis.^1^ In this report, similar clustered cysts without any scolices or post-contrast enhancement was observed but at an atypical location. The differential diagnosis for racemose variant includes tubercular meningitis; however, basilar exudates of tuberculosis (TB) are solid and are not entirely suppressed on FLAIR. Carcinomatous meningitis and neuro-sarcoid are also differentials for racemose neurocysticercosis but they are rarely cystic.

Intraventricular neurocysticercosis is usually appreciated on FLAIR imaging when CSF is suppressed. On post-contrast T1W images, intraventricular neurocysticercosis appears as an enhancing lobulated cystic lesion with or without adjoining ventriculitis. Obstructive membranes and adhesions can also develop in ventricles because of ependymitis. Additionally, complications such as ventricular dilatation and vasculitis can be seen on MRI. In this case, similar intraventricular cysts were illustrated in the atrium of the left lateral ventricle associated with an obstructive membrane in the fourth ventricle. Differential diagnosis includes ependymal cyst, choroid plexus cyst, choroid plexus papilloma, colloid cyst, arachnoid cyst and cystic metastasis. An ependymal cyst is usually unilocular, non-enhancing, behaves like CSF and displaces the choroid plexus. Choroid plexus cysts are generally bilateral and most commonly seen in the atrium of lateral ventricles within the choroid plexus glomus. The colloid cyst contains mucin and is not completely suppressed on FLAIR. Choroid plexus papilloma is usually seen below five years of age and has a solid enhancing component. Arachnoid cysts and cystic metastasis are very rarely seen in the ventricles.^16^

Newer MR sequences are now being utilised for demonstrating intraventricular and subarachnoid space neurocysticercosis. Three-dimensional CISS is a fully refocussed steady-state gradient-echo MRI sequence. This is a heavy T2W sequence that increases lesion visibility in ventricles, cisterns and subarachnoid spaces.^17^ Intraventricular neurocysticercosis, which is not well visualised on conventional MR sequences is better delineated with this sequence. Another advanced MR sequence is the 3-D spoiled gradient recalled echo sequence (SPGR), where both pre- and post-contrast T1W images are obtained. Both these sequences can identify missed obstructive membranes in intraventricular neurocysticercosis.^18^ In this report an obstructive membrane was only visualised on the 3-D SPGR sequence.

Amongst the serological tests available for neurocysticercosis, enzyme linked electro-immuno transfer blot (EITB) using lentil lectin glycoproteins (LLGPs) has a sensitivity of >90% and a specificity of 100% for detection of NCC. This assay can be performed on both serum and CSF samples and it has outperformed the classic ELISA test.^7^

Medical management of neurocysticercosis includes cysticidal drugs such as albendazole and praziquantel, steroids and antiepileptic drugs. Medical management is highly effective in the parenchymal and subarachnoid forms. Surgical management in the form of a ventriculoperitoneal shunt is required in cases of symptomatic hydrocephalus. As a result of recurrent blockage of a ventriculoperitoneal shunt, the endoscopic approach for removal of an intraventricular cyst is nowadays the preferred method of treatment.^19,20^ However, intraventricular cysts that are tightly adherent to the ependyma because of chronic ependymitis are difficult to remove endoscopically and can lead to injury.^20^

## Conclusion

Racemose neurocysticercosis and intraventricular neurocysticercosis are both rare types of neurocysticercosis. The presence of these cysts in the region of the crus of the fornix, anterior to the splenium of the corpus callosum, is very rare. For intraventricular neurocysticercosis, the atrium of the lateral ventricle is also quite an unusual location. Knowledge of the imaging appearance of racemose neurocysticercosis and the different locations where it can develop will prevent overlooking the diagnosis and excluding other differentials. Moreover, newer MR sequences like CISS and 3-D SPGR should also be acquired, in addition to conventional MR sequences, for better delineation of any cyst or membrane obstructing the ventricles and CSF spaces.
